# Treatment of Esophageal Achalasia With Sarcopenic Dysphagia by Rehabilitation and Nutritional Support: A Case Report

**DOI:** 10.7759/cureus.64529

**Published:** 2024-07-14

**Authors:** Fumiko Furukawa, Kiyohito Kakita

**Affiliations:** 1 Rehabilitation Medicine, Kyoto Ohara Memorial Hospital, Kyoto, JPN

**Keywords:** sarcopenia, oral dysphagia, nutrition therapy, rehabilitation, esophageal achalasia

## Abstract

Esophageal achalasia is a disease characterized by esophageal motor dysfunction, leading to various symptoms, including vomiting and chest pain. There is no curative treatment for this disease, and the consensus on nutritional therapy or rehabilitation is unclear. Herein, we present the case of a 90-year-old woman with symptoms of esophageal achalasia, exacerbated by secondary sarcopenia and sarcopenic dysphagia after coronavirus disease 2019 (COVID-19) pneumonia. The patient presented with chest pain and vomiting while on a soft diet, and esophagography revealed typical esophageal achalasia. Her esophageal achalasia symptoms resolved, with improvements in nutritional status, skeletal muscle mass, and physical capacity, when a combination of nutritional and comprehensive rehabilitation therapies was adopted. This case highlights that oral dysphagia is associated with worsening esophageal achalasia symptoms and that nutritional and rehabilitative interventions are effective in relieving the symptoms of achalasia in patients with esophageal achalasia and sarcopenia.

## Introduction

Esophageal achalasia is a rare disorder, and the patients present with a variety of symptoms due to impaired relaxation of the lower esophageal sphincter and lack of esophageal motility. Although rarely life-threatening, it decreases the quality of life, and cases of malnutrition and sarcopenia have been reported [1-3]. The treatment of esophageal achalasia is aimed at symptomatic improvement. There is no curative treatment, and there is no consensus regarding nutritional or rehabilitative therapy.

Sarcopenic dysphagia was first described in a study by Kuroda and Kuroda in 2012 [4], with a diagnostic algorithm published in 2017 [5]; in 2019, it was defined as an eating and swallowing disorder caused by sarcopenia of the entire body and swallowing muscles [6]. Knowledge of nutritional management and rehabilitation based on this concept is increasing. However, to our knowledge, its association with esophageal achalasia has not yet been reported.

Here, we report the case of a patient with esophageal achalasia with sarcopenia whose symptoms of esophageal achalasia worsened due to sarcopenic dysphagia but improved with nutritional therapy and rehabilitation intervention.

## Case presentation

The patient was a 90-year-old woman with a history of lumbar vertebral compression fractures a year ago. Since the fracture, her activities of daily living (ADL) gradually declined from walking alone to using a walker. Several weeks before admission, she occasionally felt regular food stuck in her throat. The patient was admitted to the acute care ward with coronavirus disease 2019 (COVID-19) pneumonia and received nutritional management with peripheral parenteral nutrition and a liquid diet due to COVID-19-related olfactory and taste disorders and loss of appetite. After five weeks of acute care, she was admitted to our hospital for rehabilitation treatment due to a decline in ADL.

On admission, she had difficulty sitting in a chair for five minutes, spent most of the day in bed, and required severe assistance with ADLs. Physical findings, nutritional status, and swallowing status at the time of admission are shown in Table 1.

**Table 1:** Physical findings, nutritional status, and swallowing function evaluation at admission The skeletal muscle mass index and maximum tongue pressure were measured using bioelectrical impedance analysis (InBody S10, InBody, Seoul, South Korea) and a tongue pressure manometer (TPM-02E; JMS Co. Ltd., Hiroshima, Japan), respectively. BMI: body mass index; MNA®-SF: mini nutritional assessment-short form; MMSE: mini-mental state examination; FIM: functional independence measure; SPPB: short physical performance battery; SMI: skeletal muscle mass index; RSST: repetitive saliva swallowing test; MWST: modified water swallowing test; FT: food test; FOIS: functional oral intake scale

Test	Result/units
Height	141 cm
Body weight	32.3 kg
BMI	16.2 kg/m^2^
MNA®-SF	3 points
Serum albumin	2.6 g/dL
MMSE	18 points
FIM motor score	25
Grip strength (right/left)	11.9 kg/10.1 kg
SPPB	0 point
SMI	4.4 kg/m^2^
RSST	3 times per 30 seconds
MWST	4 points
FT	4 points
FOIS	3
Tongue strength	19 kPa

Her body weight had decreased by 4 kg during the last six months. The patient was diagnosed with sarcopenia according to the Asian Working Group for Sarcopenia (AWGS) 2019 diagnostic criteria [7] due to low skeletal muscle mass, low muscle strength, and low physical performance. She presented with secondary sarcopenia [8] due to the COVID-19 infection and malnutrition (disease-related), inactivity, and bed rest due to isolation (activity-related). Videofluoroscopic examination of swallowing showed difficulty in transferring the food bolus from the mouth to the pharynx during the oral phase, and pharyngeal residue was observed. Based on the presence of dysphagia and generalized sarcopenia and the fact that sarcopenia is the primary cause of dysphagia, sarcopenic dysphagia was diagnosed according to the consensus diagnostic criteria for sarcopenic dysphagia [5].

**Figure 1:**
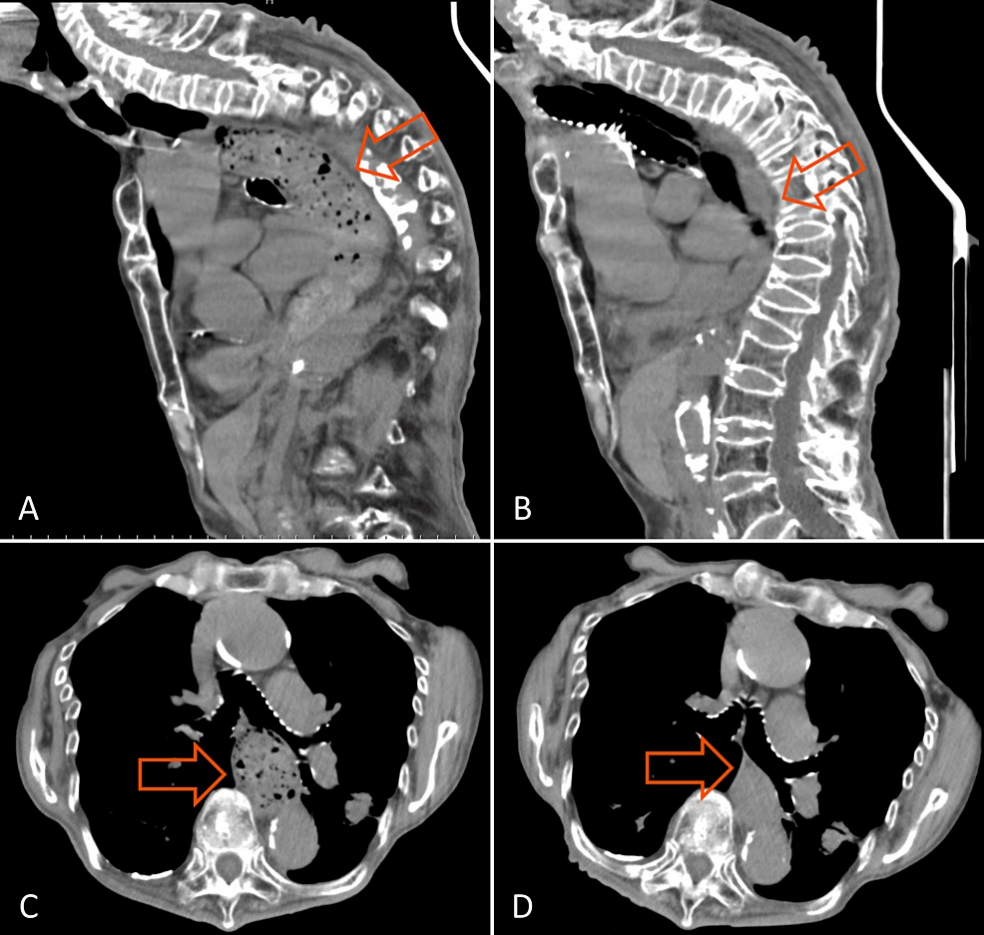
CT taken at chest pain (A, C): (A) Sagittal section showing the food mass filled the lower to upper thoracic esophagus. (C) Horizontal section at the level of the bronchial bifurcation showing the esophagus also filled with a food bolus. CT taken three hours after chest pain (B, D): (B) Sagittal section revealing most of the food mass had passed through but remained partially in the esophagus. (D) Small amount of liquid was observed in the horizontal section at the level of the bronchial bifurcation

The patient underwent rehabilitation along with nutritional therapy consisting of 30 kcal/kg/day of energy and 1.1 g/kg/day of protein based on ideal body weight (IBW). The rehabilitation intervention consisted of a usual physical and swallowing approach by physical, occupational, and speech therapists for three hours per day until discharge. The patient gradually regained her appetite as her sense of smell and taste returned and was tolerated an oral puree diet without intravenous nutrition. When she switched from a puree diet to a soft diet, she needed to chew, and severe chest pain and vomiting appeared immediately after eating. Computed tomography (CT) showed that the food mass filled the lower to the upper thoracic esophagus (Figures 1A, 1C). The chest pain spontaneously abated thereafter, and CT three hours later showed that most of the food mass had passed through but remained partially in the esophagus (Figures 1B, 1D). Subsequent esophagography revealed decreased esophageal peristalsis, residual contrast in the esophagus, and dilatation of the esophageal anastomosis, leading to the diagnosis of esophageal achalasia.

After the diagnosis of esophageal achalasia, the patient was administered a smaller amount of paste meals six times a day and kept in a wheelchair for two hours after meals. Her symptoms of esophageal achalasia diminished and disappeared along with improvement in nutritional status, skeletal muscle volume, and ADLs (Table 2).

**Table 2:** Clinical course and changes in nutritional status and ADL FIM: functional independence measure; ADL: activity of daily life

	Admission	1 month	2 months	Discharge
Body weight (kg)	32.3	32.4	33.0	33.6
Serum albumin (g/dL)	2.6	2.9	3.3	3.8
Skeletal muscle mass (kg)	12.3	12.6	Not examined	13.7
Lean body mass (kg)	26.9	27.3	Not examined	29.3
FIM-motor score	25	25	56	78
ADL	Bedbound	Reclining wheelchair	Manual wheelchair	Walk with a walker
Type of meal	Pureed	Pureed	Soft and bite-size	Regular
Number of meals (times/day)	3	6	3-5	3
Time required for meal (minutes)	50	30-40	20-30	15-20

Three months later, the patient was able to consume regular solid foods three times a day without recurrence of any esophageal achalasia symptoms and could walk with a walker. The patient was discharged to home. One-and-a-half years after discharge from the hospital, her ADLs have been maintained and esophageal achalasia has not recurred.

## Discussion

The following findings were evident in this case. First, sarcopenic dysphagia during the oral phase exacerbates esophageal achalasia. Second, a combination of nutritional and comprehensive rehabilitation therapies was effective in improving the esophageal achalasia symptoms associated with sarcopenia.

We believe that sarcopenic dysphagia, particularly during the oral phase, contributed to the exacerbation of esophageal achalasia symptoms in this patient, resulting in inadequate chewing of solid food and difficulty passing the food mass through the esophagus. The patient presented with general skeletal and swallowing-related muscle sarcopenia. Tongue pressure reflects oral-phase swallowing function [9-11] and the patient’s low tongue pressure indicates oral-phase dysphagia. The following clinical course supported the idea that sarcopenic dysphagia exacerbates esophageal achalasia. Before admission, the patient had symptoms suggestive of esophageal achalasia, such as postprandial chest discomfort but was eating a regular diet requiring mastication without vomiting. The patient developed secondary sarcopenia due to inactivity, malnutrition, and inflammation from COVID-19 infection after hospitalization and had no symptoms when eating a puree diet; however, when she switched to a soft diet requiring chewing, her symptoms of esophageal achalasia worsened. In this case, we believe that the oral-phase dysphagia resulted in inadequate processing of solid foods, which exacerbated the symptoms of esophageal achalasia.

Nutritional intervention along with rehabilitation therapy for sarcopenic dysphagia resulted in the improvement of esophageal achalasia symptoms in the present case. It has been reported that combining nutritional, rehabilitation, and physical therapy interventions for sarcopenic dysphagia may improve systemic muscle strength and function, including swallowing muscles [6,12-15], which is consistent with the results of this case. In the present case, the improvement in general endurance that was hindered by sarcopenia was also important. Fatigue during meals due to decreased endurance was augmented by the longer time required to eat due to decreased tongue movement and chewing ability caused by sarcopenic dysphagia. This exacerbated fatigue leads to further inadequate tongue movements and chewing, resulting in a negative cycle of worsening achalasia. We believe that the improvement in general endurance, in addition to the improvement in swallowing function, was effective in reducing the symptoms of esophageal achalasia, because the patient was able to process solid food into a form that could easily pass through the esophagus in a shorter time without causing fatigue. This result suggests that not only improvement in swallowing function but also general endurance is important for the improvement of esophageal achalasia symptoms caused by sarcopenic dysphagia.

We set nutritional goals to improve sarcopenia and malnutrition in this case at 30 kcal/kg/day of energy and 1.1 g/kg/day of protein, as determined using IBW. In patients with sarcopenic dysphagia, nutritional supplementation of 25-35 kcal/kg/day and 1.0-1.2 g/kg/day of protein based on IBW is recommended which causes increasing body weight and improving ADL and swallowing function of patients with sarcopenic dysphagia [12-15]. Initially, the patient was required to eat smaller amounts of frequent meals to meet the nutritional requirements while preventing esophageal achalasia symptoms. By reducing the amount of food consumed per meal, the patient was able to masticate steadily without feeling tired, and the increased frequency of meals increased the opportunity to chew food, which may have had a positive effect on the swallowing muscles. The symptoms of esophageal achalasia disappeared with improvements in nutritional status, skeletal muscle mass, and physical performance.

In the present case, the impact of COVID-19 on the symptoms of esophageal achalasia and the current swallowing disorder is unclear. Since the COVID-19 pandemic, case reports of esophageal achalasia triggered by COVID-19 have accumulated, suggesting the involvement of the virus in the onset of esophageal achalasia symptoms [16-19], the impact of COVID-19 on the worsening of esophageal achalasia symptoms is unknown. In patients with esophageal obstructive disease, such as esophageal achalasia, whose symptoms are exacerbated after disease- or activity-related sarcopenia, a combination of nutritional and rehabilitation interventions may be effective in improving exacerbated symptoms. Research on the relationship between esophageal achalasia and the new coronavirus is currently ongoing, and future reports, including long-term sequelae, are warranted.

The limitations of this report are as follows. First, the post-discharge follow-up period was short (18 months after discharge) and the long-term sustainability of the improvement is unknown; therefore, further observation is needed to determine the long-term effects of the treatment. Second, physiological evidence to explain the interrelationship between sarcopenic dysphagia and esophageal achalasia was lacking. Third, because this case report focused on one patient, we cannot rule out the possibility that the improvement in esophageal achalasia, in this case, was due to the natural course or other potential confounding factors, rather than rehabilitation and nutritional interventions. We believe that further accumulation and examination of cases is necessary to validate our findings in this report.

## Conclusions

This case highlights that oral dysphagia is associated with worsening esophageal achalasia symptoms and that nutritional and rehabilitative interventions are effective in relieving the symptoms of achalasia in patients with esophageal achalasia and sarcopenia.

Multiple factors, including dysphagia and decreased general endurance, may have contributed to the worsening of esophageal achalasia symptoms in this patient, and the integrated effects of improved nutritional status and physical function through a combination of nutritional and rehabilitation interventions may have helped the patient achieve favorable outcomes. We expect nutritional therapy and rehabilitation to also be effective in patients with oral-stage dysphagia. This was a single-case report, and further studies are needed to determine the usefulness of nutritional rehabilitation for patients with untreated esophageal achalasia with sarcopenia.
